# Medical News and Miscellany

**Published:** 1889-06

**Authors:** 


### MEDICAL NEWS AND MISCELLANY.

DR. F. F. FOUTS, of Floresville, was assassinated by negroes on the 17th instant.

THE railroads have *all refused* to give the American Medical Assciation Delegates reduced rates to Newport.

WE regret to learn that Dr. J. M. Ball, of New Boston, is laid up with rheumatism at Hot Springs, Arkansas.

DR. S. FARROW STYLES, of Brenham, we regret to learn, was thrown from his buggy recently, and dangerously injured.

FOR SALE.—A well established practice; a good residence with fine improvements in one of the healthiest and best towns in the State. For information address editor of this journal.

DR. S. FARROW STYLES has removed from Independence to Brenham. The doctor has been sick since the San Antonio meeting, but we are pleased to state that he has recovered and resumed work.

THE AMERICAN PUBLIC HEALTH ASSOCIATION will meet in Brooklyn, New York, October 22, 23, 24 and 25, 1889. Dr. H. A. Johnson, Chicago, President; Dr. Irvin A. Watson, Concord, New Hampshire, Secretary.

REMOVAL—Dr. T. Farrow Styles, from Independence to Brenham. Dr. J. W. Cartwright, from Decatur to Amarillo. Dr. D B. McGee, from Hinckley to May. Dr. W. B. Watkins, from Wills Point to San Angelo. Dr. A. M. Womack, from Meridian to Mount Vernon.

DR. A. M. WOMACK, of Mt. Vernon, Texas. We regret exceedingly to learn that this worthy brother and long time practitioner has had a series of almost unprecedented misfortune—family affliction, loss of property, drought experience, and finally an attack of partial paralysis. The hand of affliction has been heavily laid upon him. But it is gratifying to learn that he has stood up bravely under his reverses, and, his health being better, he is again battling with the world. We wish him better luck. The doctor has our sincerest sympathy.

---

A LITTLE GIRL'S DIAGNOSIS.—A little girl startled her teacher and the pupils on entering the school room, by the remark, "Sister can't come to-day, she's got the glanders!"

"Glanders," exclaimed the teacher, "why, horses have the glanders!"

"I don't care," said the little girl, "I know she's got 'em, cause her glan's is all swelled up!"

---

FOR SALE AT A BARGAIN.—A good home and practice; a good five room dwelling on a four acre lot, well improved, a good kitchen and dining room, servant's room, and good out houses; fine young orchard and vineyard, good brick cistern; at flourishing railroad depot. Postoffice and express office, also good school. Address, DR. WM. OSBORNE,
Axtell, McLennan, county, Texas.

---

THE Philadelphia Medical Times, The Medical Register and The Dietetic Gazette have united, and will hereafter be published as a weekly, devoted to general medicine, with a quarterly devoted to dietectics.

The Journal will be under the charge of Dr. William F. Waugh. It will be practical in character, and devoted to the interests of practitioners.

Dr. Waugh has been in the editorial harness for over four years; first with the Medical Morld, then with the Philadelphia Medical Times, which he has edited since October, 1887.

The editorial labors will be shared by the members of the American Medical Press Association, under whose auspices the

Journal is issued. The office is at No. 1725 Arch street, Philadelphia.

---

DEATH OF DR. ASHWORTH.—It is with much pain and regret we observe in the *Atlanta Medical and Surgical Journal* notice of the death of Dr. A. B. Ashworth, editor and manager of that journal. He died May 18th, ult. Dr. Ashworth graduated in the Atlanta Medical College in '86, and entered the editorial office of the *Journal* as assistant to Dr. Gray. On the death of that most estimable gentleman, Dr. Ashworth succeeded to the editorial and business management of the *Journal* which he conducted with marked ability. He had also succeeded Dr. Gray as Secretary of the Georgia Medical Association, filling the office one term. His loss is much deplored and will be a heavy blow to the *Journal*. The *Journal* for June contains a handsome cut of the deceased. He was a very young man, apparently, for so responsible positions—age not given in the obituary notice.

---

A SHORT time ago the Statesman published a special from Decatur, Texas, announcing the death of Dr. Cartwright, of that city. The Secretary of the State Medical Association made a minute of it, and in his report at San Antonio read out the name as among the members who had died during the year. Recently we learned that Dr. Cartwright had removed to Amarillo, (and not to heaven, as we had inferred from the Statesman's article.) Instituting inquiry, we were much gratified to learn that the doctor still lives, and that the telegram above referred to had reference to a younger brother of the doctor's who was familiarly called "Doc." Cartwright. We are pleased to make the correction.

Dr. Cartwright has located at Amarillo, away up in the Panhandle, and has purchassed a ranch near by, which divides his attention with his practice. He has ordered the JOURNAL forwarded to him, and says he cannot do without it. The doctor speaks enthusiastically of the delightful climate, and says "the atmosphere contains more oxygen to the square inch than any country on earth. Come and spend a season with us, and inflate your lungs." Ah, if we just only could!

---

THE following new appointments have been made at the New York Polyclinic: Dr. Thomas R. Pooley, Surgeon-in-Chief of the New Amsterdam Eye and Ear Hospital; Ophthalmic Surgeon to the Sheltering Arms; Consulting Ophthalmologist to St. Bartholomew's Hospital; Professor of Cphthalmology.

Dr. B. Sachs, Neurologist to the Montifiore Home for Chronic Invalids; Professor of Neurology.

Dr. L. Emmett Holt, Visiting Physician to the New York Infant Asylum; Consulting Physician to the Hospital for Ruptured and Crippled; Professor of Diseases of Children.

Dr. August Seibert, Physician to the Children's Department of the German Dispensary; Professor of Diseases of Children.

Dr. H. Marion Sims, Gynecologist to St. Elizabeth's Hospital, and New York Infant Asylum; Professor of Gynecology.

Dr. Wm. H. Fluhrer, Surgeon to Mt. Sinai and Bellevue Hospitals; Professor of Genito-Urinary Surgery.

The Polyclinic has increased its hospital facilities by the purchase of a large building immediately adjoining its original property, and after making necessary changes will furnish and have it open by Sept. 16th, when the regular session will commence.

---

